# Lichen planus pemphigoides: A unique form of bullous and lichenoid eruptions secondary to nivolumab

**DOI:** 10.1111/dth.15432

**Published:** 2022-03-15

**Authors:** Rohan R. Shah, Chinmoy Bhate, Amanda Hernandez, Chin Hung Ho

**Affiliations:** ^1^ Department of Dermatology Rutgers New Jersey Medical School Newark New Jersey USA; ^2^ Department of Dermatology ProPath Dallas Texas USA; ^3^ St. Lawrence Health System Clarkson University Postdam New York USA

**Keywords:** cutaneous adverse reactions to chemotherapy, drug eruptions, nivolumab, PD‐1 inhibitor

## Abstract

The widespread use of PD‐1 inhibitors to treat various solid tumors has brought certain challenges for the clinician, including immune‐related adverse events (irAEs). Cutaneous toxicities are among the most observed irAEs. Bullous and lichenoid dermatoses following PD‐1 inhibitor therapy have been described. Here we report a novel case of lichen planus pemphigoides, featuring characteristics of both bullous pemphigoid and lichen planus, in a patient treated with nivolumab for renal cell carcinoma. We subsequently review all three cutaneous conditions which may arise in the context of PD‐1 inhibitor therapy.

## 1 INTRODUCTION

Anti‐programmed death‐1 (PD‐1) antibody targets checkpoint inhibitors to enhance immune response towards cancer cells. This form of immunotherapy is growing in popularity for patients with various malignancies.^1^ PD‐1 inhibitors have improved outcomes in melanoma, non‐small cell lung cancer (NSCLC), urothelial cancer, and renal cell carcinoma patients.^2^ However, cutaneous immune‐related adverse events (irAEs) may occur in up to 34% of patients treated with PD‐1 inhibitors.^3^ Although rare, bullous pemphigoid (BP) has been increasingly reported following PD‐1 inhibitors. Lichen planus (LP) and lichen planus pemphigoides (LPP) have also been reported but less frequently.

BP is characterized by tissue‐bound and circulating autoantibodies directed against hemidesmosome proteins, BP antigen 180 and 230.^4^ BP development following administration of PD‐1 inhibitors may lead to discontinuation of PD‐1/PD‐L1 inhibitor therapy in more than 70% of patients.^5^ Interestingly, BP development as an adverse skin reaction may act as a marker for extent of tumor progression and efficacy of the PD‐1 inhibitor in treating the underlying malignancy.^6^

LP is idiopathic; the prevailing theory is that a T‐cell‐mediated autoimmune disease follows exposure to a virus, drug, or allergen.^7^ LPP has characteristics of LP and BP. Like LP, LPP is idiopathic, but immunofluorescence assays have identified anti‐basement membrane antibodies against the C‐terminal region of the BP180 protein, the common pathogenic antigen in BP.^8^

## 2 CASE

**FIGURE 1:**
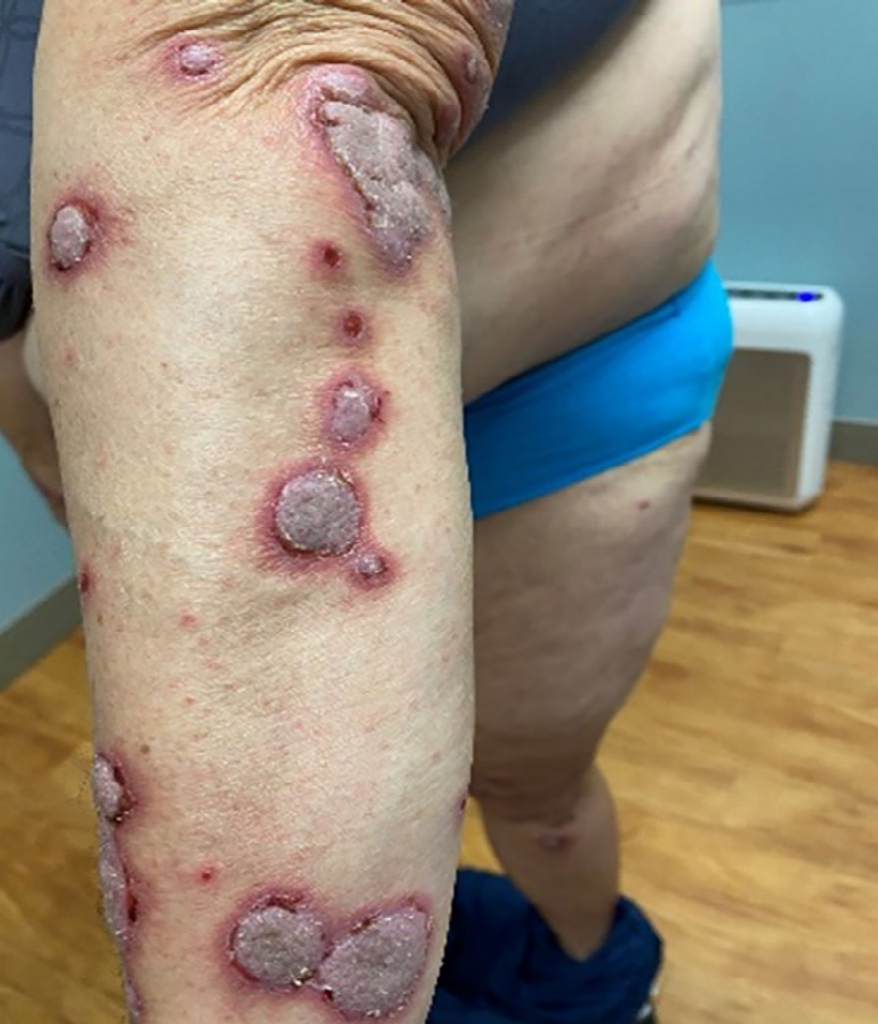
Violaceous plaques with polygonal configuration affecting flexural area and dorsal aspect of the arm with tense blisters and erosions

**FIGURE 2:**
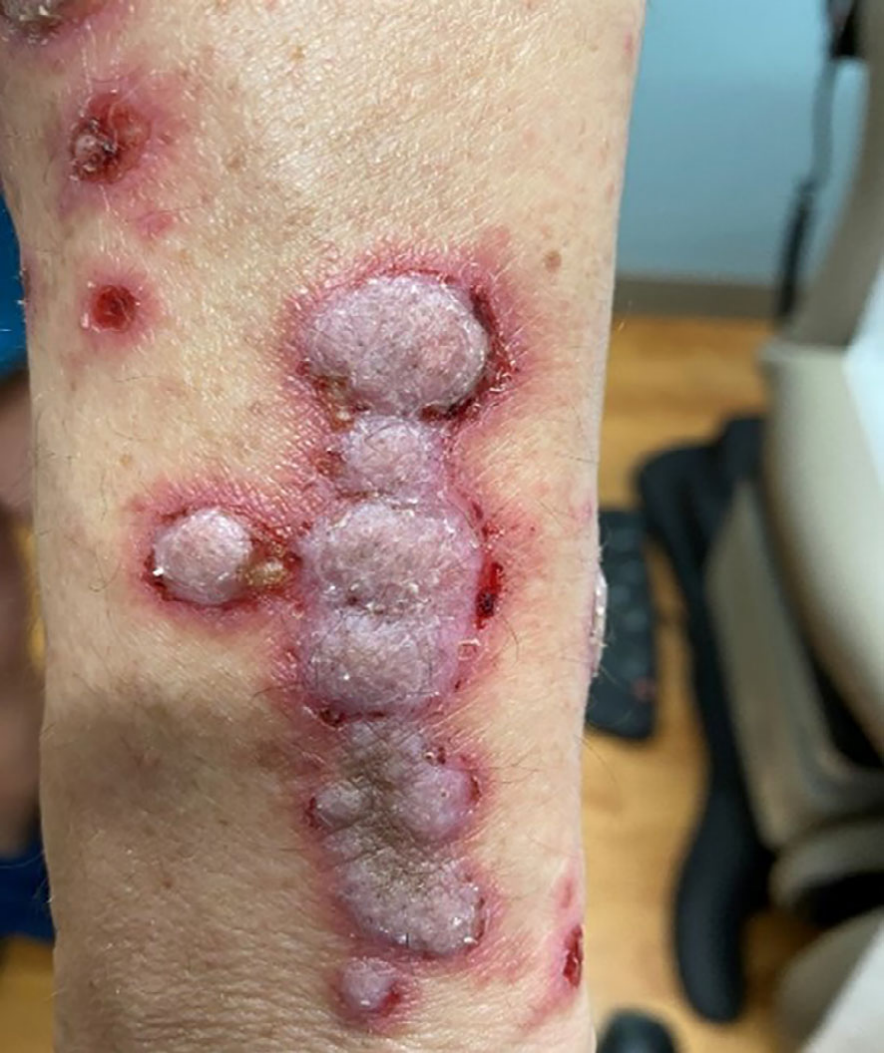
Linearly‐oriented thickened erythematous to violaceous plaques along an extremity

**FIGURE 3:**
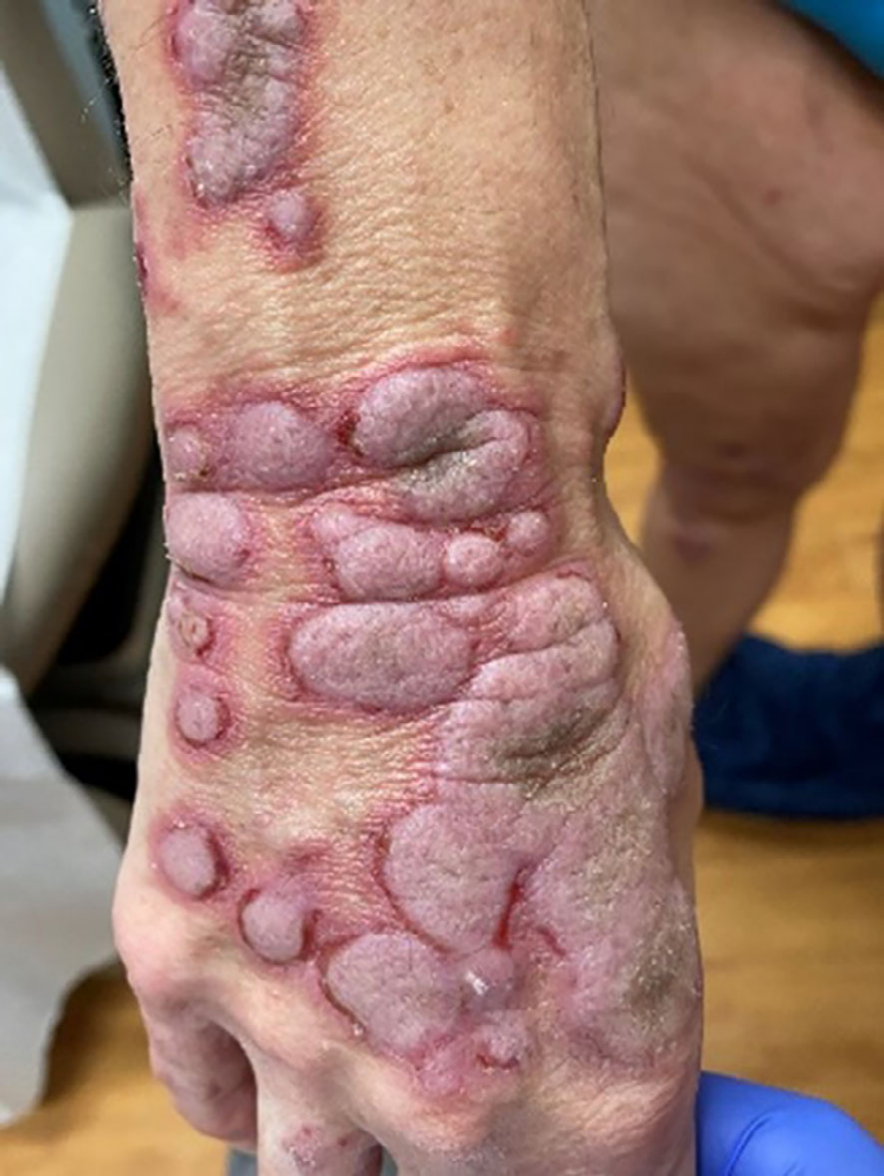
Erythematous, verrucous‐like plaques on the dorsal aspect of the hand with tense blisters and erosions

A 58‐year‐old woman with a medical history of hypercholesteremia, obesity, and vitamin D deficiency was diagnosed with renal cell carcinoma and subsequently received nivolumab treatment. Over the next 4 months, the patient developed bullae along with thickened, pruritic, and painful plaques (Figures 1, 2, 3). The patient was initially treated with topical clobetasol cream, systemic corticosteroids, and oral pregabalin. Additionally, nivolumab was discontinued with partial improvement within a month; however, persistence of manifestations led to dermatologic evaluation 3 months after initial onset of the rash. Other medications at the time of dermatologic evaluation included sertraline, magnesium, vitamin D3, calcium, and biotin. The clinical differential diagnosis included epidermolysis bullosa, dermatitis herpetiformis, paraneoplastic pemphigus, and a verrucous fungal infection. Skin biopsy was performed for permanent section evaluation and for direct immunofluorescence testing.

**FIGURE 4:**
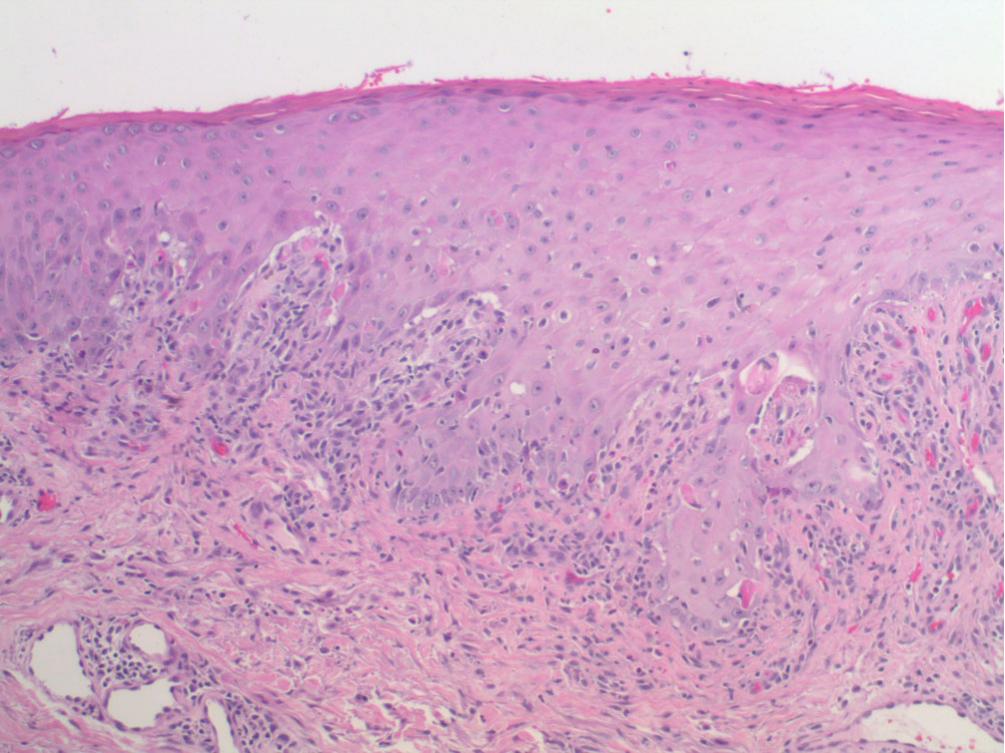
There is acanthosis of the epidermis. A band‐like inflammatory infiltrate composed predominantly of lymphocytes with scattered eosinophils is seen in the papillary dermis. There is vacuolar degeneration of the basal layer of the epidermis and scattered dyskeratotic keratinocytes (H&E, 10×)

**FIGURE 5:**
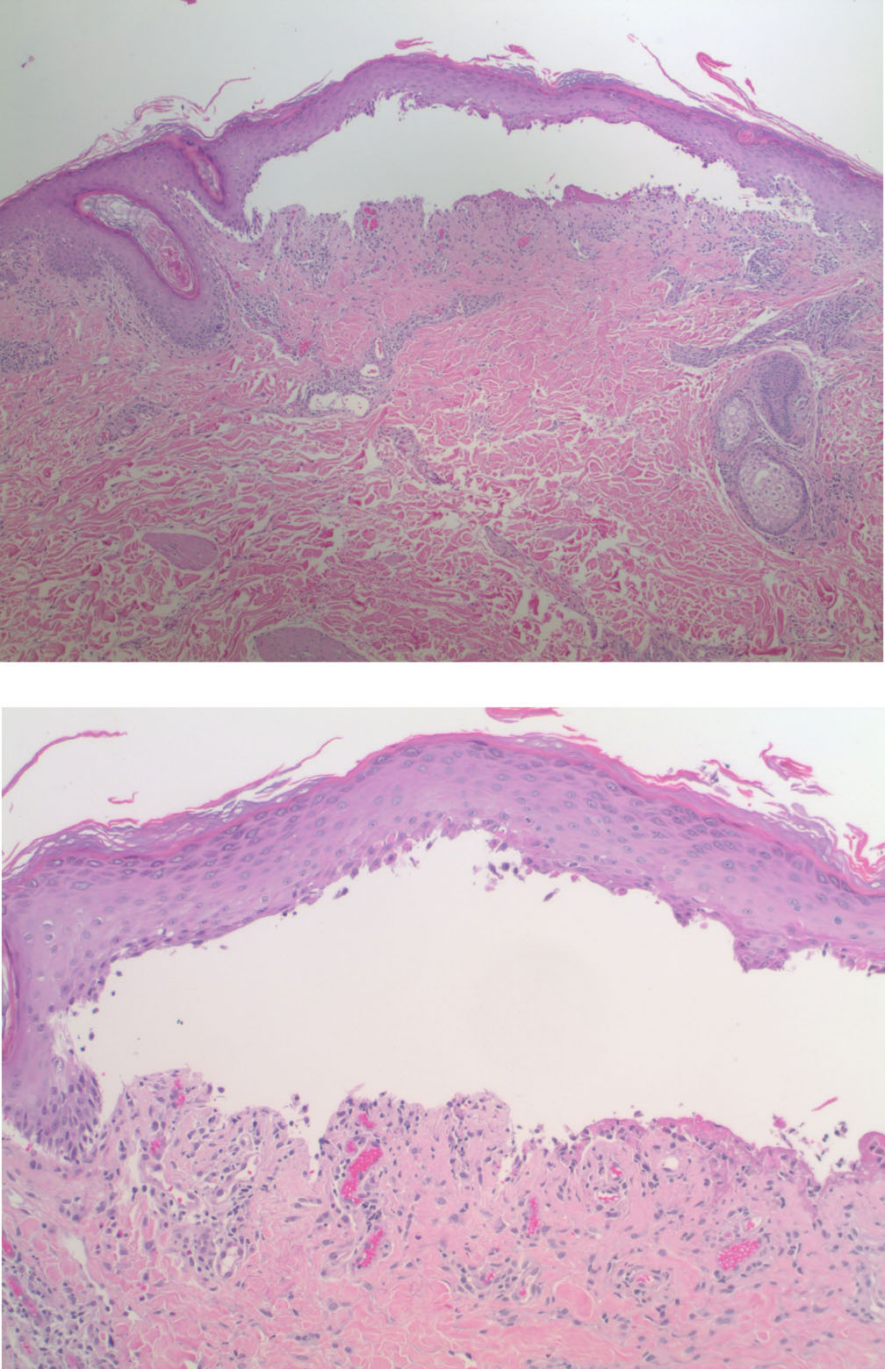
There is a subepidermal blister. Within the superficial dermis, there is a sparse lichenoid and perivascular inflammatory infiltrate with scattered eosinophils (H&E, 4×, 10×)

**FIGURE 6:**
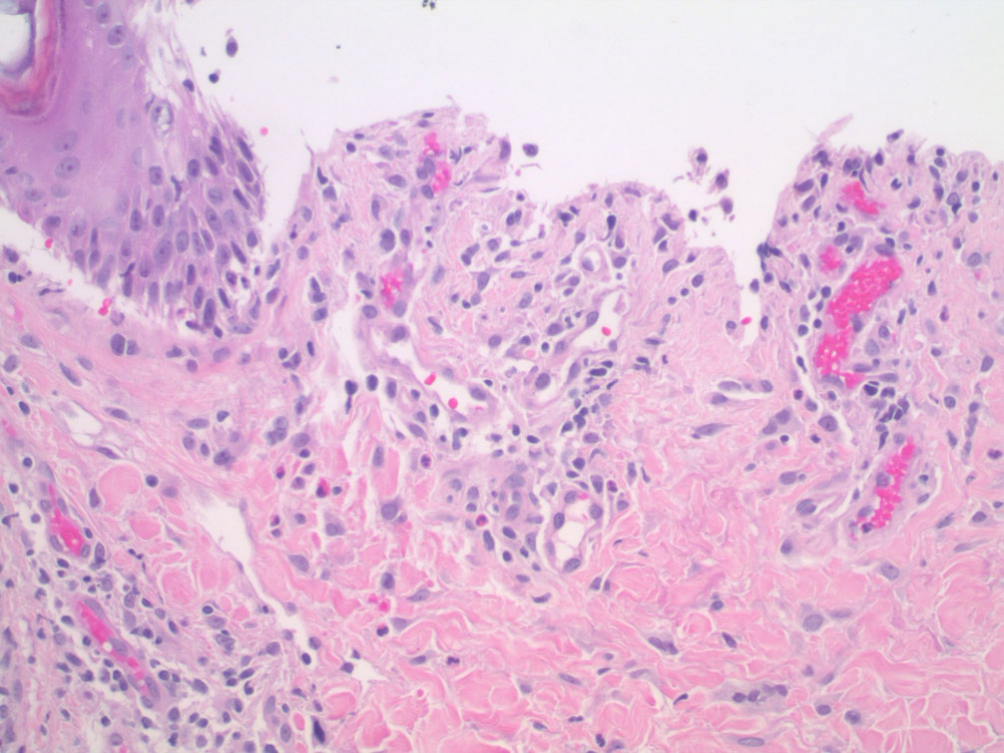
Higher power image showing the floor of the blister and the inflammatory infiltrate with scattered eosinophils (H&E, 20×)

**FIGURE 7:**
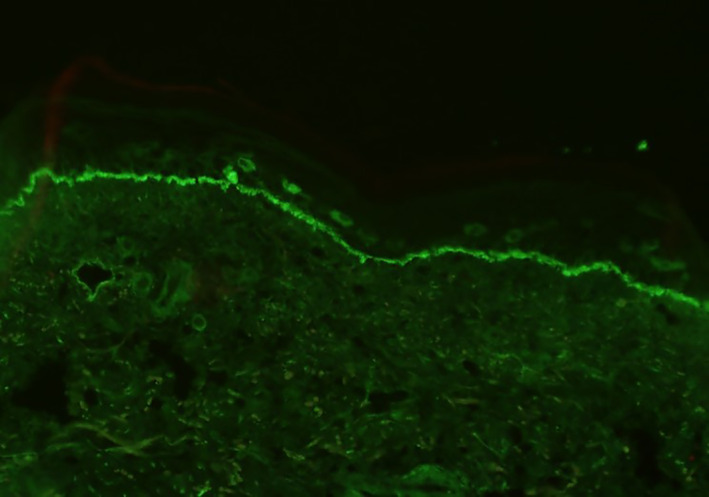
Direct immunofluorescence revealing linear C3 in the basement membrane zone

Histopathologic examination showed acanthosis of the epidermis along with a band‐like inflammatory infiltrate composed predominantly of lymphocytes with scattered eosinophils in the papillary dermis (Figure 4). There was vacuolar degeneration of the basal layer of the epidermis and scattered dyskeratotic keratinocytes. In addition, there was a subepidermal blister with an underlying sparse dermal perivascular infiltrate containing scattered eosinophils (Figures 5 and 6). Direct immunofluorescence testing demonstrated a linear deposition of C3 in the basement membrane zone (Figure 7). A diagnosis of nivolumab‐induced LPP was rendered.

## 3 DISCUSSION

### 3.1 BP in association with PD‐1 inhibitor therapy

BP in oncologic patients may be paraneoplastic, drug‐induced, secondary to cancer therapy, or idiopathic. Increasing usage of immunotherapy for cancer management has resulted in an increased incidence of BP as a cutaneous toxicity.^9^

The characteristics of the underlying malignancy may also contribute to BP development following PD‐1 inhibitor therapy. A study evaluating general cutaneous side effects of anti‐PD‐1 therapy reported 11 patients with BP; primary tumors were either melanoma (5), non‐small cell lung carcinoma (2), urothelial carcinoma (2), or head and neck squamous cell carcinoma (1).^10^ Another study also found renal cell carcinoma to be associated with BP following PD‐1 therapy. Additionally, two 70‐year‐old patients with stage IV melanoma and lung metastases developed BP between 9 and 12 months following the usage of PD‐1 inhibitors.^11^

Unlike other dermatologic irAEs which occur early in treatment, immunotherapy‐induced bullous dermatoses demonstrate latency beyond the first treatment cycle. Often, mild and localized pruritus is the only clinical symptom before quickly progressing to hemorrhagic blisters on the chest, abdomen, thighs, and upper arms.^9^ The average time to diagnosis in nine reported cases was 9.4 months.^12^ A review of 21 cases with PD‐1‐induced BP found that nine were associated with nivolumab in which the median time to cutaneous toxicity and bullae formation was 12 weeks.^5^ In addition to a delay in BP onset following PD‐1 inhibitor therapy, a lag time exists to resolution following discontinuation of the treatment. In one instance, de novo bullae continued to form for 10 months after cessation, suggesting that discontinuation of PD‐1 treatment does not immediately halt BP lesion formation.^13^ This lag time to progression and resolution must be considered when managing BP and similar conditions.

The reason for BP development in patients receiving PD‐1 inhibitors is unknown. One theory specific to melanoma patients suggests that malignant melanocytic tumor cells express BP180 while benign melanocytes do not.^14^ A majority of cases reporting BP following PD‐1 immunotherapy have been stage IV, correlating with the setting in which these agents are most often used.^11^ However, aside from identifying the type of cancers most commonly associated with PD‐1 inhibitor induced BP, it may be noteworthy to determine whether a correlation exists between BP and severity of cancer.

Pembrolizumab and nivolumab are the more commonly used PD‐1 inhibitors.^5^ A retrospective study using the Research on Adverse Drug Events and Reports Program methodology to search the Food and Drug Administration (FDA) Adverse Event Reporting System (FAERS) from the first FDA approval date to the first quarter of 2018 found nivolumab to be more associated with cases of BP followed by pembrolizumab.^15^ The severity of autoimmunity induced by PD‐1 inhibitors is variable but may correlate with extent of antitumor response.^16^ In certain cases, multiple autoimmune disorders were seen following anti‐PD‐1 therapy. For instance, one patient with stage IV melanoma developed both BP and vitiligo after two infusions of nivolumab.^16^ The extent of pemphigoid was also more pervasive with oral mucosa involvement. Nevertheless, the cancer regressed. This is further illustrated by a report describing 15 cases of BP following PD‐1 therapy, in which a stronger autoimmune response correlated with extent of antitumor response and regression of metastasis. Of the 15 cases, two had complete or partial response while six had stable disease.^6^

### 3.2 Lichenoid dermatoses in association with PD‐1 inhibitor therapy

LP is a chronic, immune‐mediated, inflammatory disease affecting the skin, nails, eyes, mucous membranes, and urinary tract.^17^ Drug‐induced LP has been associated with various agents including antimalarials, NSAIDs, beta‐blockers, thiazide diuretics, and in recent years, PD‐1 inhibitors.^18^ Shi et al.^19^ reported that of 17 patients who underwent biopsy for bullous lesions following PD‐1 inhibitor therapy, 16 had features of lichenoid interface dermatitis, suggesting that there is a distinct cutaneous lichenoid eruption associated with anti‐programmed cell death 1 therapy.^19^

A review of multiple cases reporting LP following PD‐1 inhibitor therapy found malignant melanoma and NSCLC to be the leading malignancies for starting PD‐1 inhibitors. The average age of LP development in this context was 66.6 years with a balanced sex distribution. Varying severities have been reported following PD‐1 inhibitors depending on concurrent treatment for underlying malignancy.^20^ For instance, combinational therapy of radiation and nivolumab has resulted in multiple, erosive LP. The initial eruptions only became erosive after radiation therapy. Notably, the erosive LP appeared within 4 weeks following radiation and appeared on extremities which had not been irradiated. Since previous reports have shown that LP appears approximately 17 weeks after PD‐1 immunotherapy, it is likely that the radiation expedited pathogenesis, perhaps by activating auto‐reactive T cells.^7^

LPP is a rare subepidermal blistering disorder with characteristics of LP and BP. Bullae of LPP may appear in areas of grossly lichenoid eruptions, or independently on uninvolved skin.^8^ To our knowledge, seven cases have reported LPP following PD‐1 inhibitor therapy. While one previous report does indicate a LPP case following pembrolizumab, a separate study found this conclusion unlikely since clinical and histological findings lacked blisters of LP lesions.^20^ In one case, an 87‐year‐old female with NSCLC was diagnosed with LPP following 9 cycles of nivolumab. Another case of a 57‐year‐old male with NSCLC had LPP after 3 months of nivolumab treatment. In both cases, discontinuation of nivolumab and administration of systemic steroids led to resolution of the LPP within 2 weeks, with clinically stable malignancy.^21^

### 3.3 Treatments

Treatment options for PD‐1 inhibitor induced BP, LP, and LPP focus on arresting development of new lesions and controlling symptoms while attempting to limit cancer progression. For most cases of bullous dermatoses in this setting, topical or systemic steroids are routinely utilized, depending on severity.^22^ Rebound flares of BP following oral steroid tapers have been described.^23^ Systemic corticosteroids are often used when topical therapies demonstrate minimal improvement. Oral nicotinamide and tetracyclines have also demonstrated positive effects in mild to moderate cases of BP, specifcally.^17^ Other immunosuppressives, including azathioprine, mycophenolate, rituximab, and omalizumab have been used for refractory cases after discontinuation of immunotherapy in BP.^24^ For LPP, a review of reported treatment outcomes for LPP since 2000 suggested that a combination of topical steroids, prednisolone pulse therapy, and acitretin is sufficient to induce remission of blistering within 3 months and disappearance of lesions within 1 year.^25^

The discontinuation, resumption and/or dosing of PD‐1 inhibitor therapy following the onset of BP, LP, and LPP remain unclear, especially in patients whose malignancy is immunotherapy‐responsive. One patient showed improvement in BP after switching PD‐1 inhibitors from nivolumab to pembrolizumab.^21^ Another case demonstrated recurrent and more severe BP after a higher dose of nivolumab was used.^22^

Awareness of BP, LP, and LPP following PD‐1 inhibition is critical as immunotherapy becomes more commonly utilized. Further observation is required to identify the underlying mechanisms of these processes and to assess options which may mitigate their course.

## CONFLICT OF INTEREST

The authors have no relevant financial conflicts of interest to disclose.

## AUTHOR CONTRIBUTIONS

Rohan R. Shah: drafted the manuscript, edited the manuscript, and worked with other authors in formulating ideas for the topic. Chinmoy Bhate: helped draft the manuscript, edited the manuscript, and developed key ideas to discuss in the paper. Amanda Hernandez: edited the manuscript, provided the dermatopathology images, and helped formulate ideas discussed in manuscript. Chin Hung Ho: edited the manuscript and helped formulate ideas discussed in manuscript.

## INFORMED CONSENT

Informed consent was provided and obtained from our patient.

## Data Availability

There is no data in our paper. All the information is derived from existing literature.
